# Regulation of the epithelial to mesenchymal transition and metastasis by Raf kinase inhibitory protein-dependent Notch1 activity

**DOI:** 10.18632/oncotarget.6728

**Published:** 2015-12-22

**Authors:** Hae Sook Noh, Young-Sool Hah, Ji Hye Ha, Min Young Kang, Sahib Zada, Sun Young Rha, Sang Soo Kang, Hyun Joon Kim, Jae-Yong Park, June-Ho Byun, Jong Ryeal Hahm, Jeong Kyu Shin, Sang-Ho Jeong, Young-Joon Lee, Deok Ryong Kim

**Affiliations:** ^1^ Department of Biochemistry and Convergence Medical Sciences, Institute of Health Sciences, Gyeongsang National University School of Medicine, JinJu, Republic of Korea; ^2^ Biomedical Research Institute of Gyeongsang National Hospital, Gyeongsang National University, JinJu, Republic of Korea; ^3^ Department of Obstetrics and Gynecology, Institute of Health Sciences, Gyeongsang National University School of Medicine, JinJu, Republic of Korea; ^4^ Department of Internal Medicine, Yonsei Cancer Center, Songang Institute for Cancer Research, Yonsei University College of Medicine, Seoul, Republic of Korea; ^5^ Department of Anatomy and Convergence Medical Sciences, Institute of Health Sciences, Gyeongsang National University School of Medicine, JinJu, Republic of Korea; ^6^ School of Biosystem and Biomedical Science, College of Health Science, Korea University, Seoul, Republic of Korea; ^7^ Department of Oral and Maxillofacial Surgery, Institute of Health Sciences, Gyeongsang National University School of Medicine, JinJu, Republic of Korea; ^8^ Department of Internal Medicine, Institute of Health Sciences, Gyeongsang National University School of Medicine, JinJu, Republic of Korea; ^9^ Department of Surgery, Institute of Health Sciences, Gyeongsang National University School of Medicine, JinJu, Republic of Korea

**Keywords:** RKIP, EMT, metastasis, Notch1, ERK

## Abstract

Raf kinase inhibitory protein (RKIP), an endogenous inhibitor of the extracellular signal-regulated kinase (ERK) pathway, has been implicated as a suppressor of metastasis and a prognostic marker in cancers. However, how RKIP acts as a suppressor during metastasis is not fully understood. Here, we show that RKIP activity in cervical and stomach cancer is inversely correlated with endogenous levels of the Notch1 intracellular domain (NICD), which stimulates the epithelial to mesenchymal transition (EMT) and metastasis. The levels of RKIP were significantly decreased in tumor tissues compared to normal tissues, whereas NICD levels were increased. Overexpression of RKIP in several cell lines resulted in a dramatic decrease of NICD and subsequent inhibition of several mesenchymal markers, such as vimentin, N-cadherin, and Snail. In contrast, knockdown of RKIP exhibited opposite results both *in vitro* and *in vivo* using mouse models. Nevertheless, knockdown of Notch1 in cancer cells had no effect on the expression of RKIP, suggesting that RKIP is likely an upstream regulator of the Notch1 pathway. We also found that RKIP directly interacts with Notch1 but has no influence on the intracellular level of the γ-secretase complex that is necessary for Notch1 activation. These data suggest that RKIP plays a distinct role in activation of Notch1 during EMT and metastasis, providing a new target for cancer treatment.

## INTRODUCTION

Tumor metastasis is a multistep process that begins with the separation of cancer cells from the primary tumor followed by local invasion, intravasation into the bloodstream, extravasation, and final colonization at distant organs. Metastasis is the major cause of cancer-associated death and poor prognosis in humans. Each step of tumor metastasis requires specific cell-cell interactions and many different signaling pathways [1, 2]. Before the metastatic progression of a tumor begins, a cancer cell commits to a transition from an epithelial to a mesenchymal phenotype; this process is called the epithelial-mesenchymal transition (EMT). It allows cancer cells to be highly migratory during metastasis [3, 4]. The EMT process coincides with the simultaneous loss of epithelial markers, such as E-cadherin, and the acquisition of mesenchymal markers, such as N-cadherin and vimentin, by activating the transcriptional factors, Snail, Slug, Twist, and ZEB1/ZEB2 [5, 6].

Raf kinase inhibitory protein (RKIP) is an inhibitory protein in the Raf/mitogen-activated protein kinase (MAPK, or MEK)/extracellular signal-regulated kinase (ERK) pathway via a direct interaction with Raf-1 [7]. Thus, RKIP is a key signaling modulator in the MAPK pathway that controls cell fate under many cellular conditions [8]. RKIP also controls nuclear factor kappa-light-chain-enhancer of activated B cells (NF-*κ*B) activity by suppressing the activity of inhibitors of *κ*B kinase α and β (IKKα and IKKβ, respectively), NF-*κ*B inducing kinase (NIK), and transforming growth factor-β-activated kinase 1 (TAK1) [9, 10]. In addition to these cellular functions, RKIP plays an important role in suppressing tumor progression and metastasis through coordination of these intracellular signals [11–13]; thus, it is known as a suppressor of metastasis and a good prognostic marker for identifying disease-free survival in cancer patients. According to many previous reports, RKIP is severely downregulated in many human primary cancers, such as the highly metastatic prostate [14–16], breast [17, 18], colon [19–21], ovarian [22], and cervical cancers [23, 24], and glioma [25]. These *in vivo* data complemented by *in vitro* studies suggest that RKIP could inhibit both the signaling pathway that governs EMT and the multistep process of metastasis from migration/invasion to homing. However, the detailed role of RKIP in the inhibitory mechanisms underlying these processes still remains to be discovered.

Activation of Notch signaling is a crucial step for tumor survival and progression [26, 27]. Indeed, the Notch pathway is aberrantly activated in many solid tumors, including cervical, head and neck, liver, lung, prostate, and breast cancer, and its activation is functionally associated with metastasis in these tumors [28]. Notch, a transmembrane receptor protein, is composed of four distinct family members (Notch1-4) in humans. In particular, ligand binding to Notch1 causes release of the Notch1 intracellular domain (NICD) via the proteolytic activity of the γ-secretase complex, which is composed of a catalytic subunit (Presenilin-1 or Presenilin-2) and accessory subunits (Presenilin enhancer 2 (PEN2), Aph1, and Nicastrin) [29, 30]. The NICD fragment subsequently translocates into the nucleus and forms a transcriptional complex with other factors, including mastermind-like protein (Maml) and C-promoting binding factor 1 (CBF1)/Suppressor of hairless/Lag-1 (CSL), resulting in the transcriptional activation of EMT-related genes, such as Slug or Snail [26, 27]. Therefore, activation of Notch1 (production of NICD) has been implicated in tumorigenesis, proliferation, and survival of several cancer cells. Moreover, NICD is associated with poor survival in patients with breast cancer and non-small cell lung cancer [31–35]. Some recent studies suggest that activation of Notch1 signaling promotes cancer metastasis by stimulating EMT via Snail- or Slug-mediated repression of E-cadherin in cancer cells [31, 33].

In this study, we aimed to understand the molecular mechanisms governing RKIP-dependent Notch1 activation in tumor progression using overexpression or knockdown of RKIP in cancer cells. We found that RKIP directly binds to Notch1 and prevents the proteolytic cleavage of Notch1 by γ-secretase. As a result, RKIP suppresses NICD production and inhibits NICD-mediated cell invasion and migration during metastasis. We also demonstrate that RKIP expression is inversely related to NICD activation in the cervical and stomach tissues of human cancer patients.

## RESULTS

### RKIP overexpression suppresses activation of Notch signaling in lung and cervical cancer cell lines

**Figure 1:**
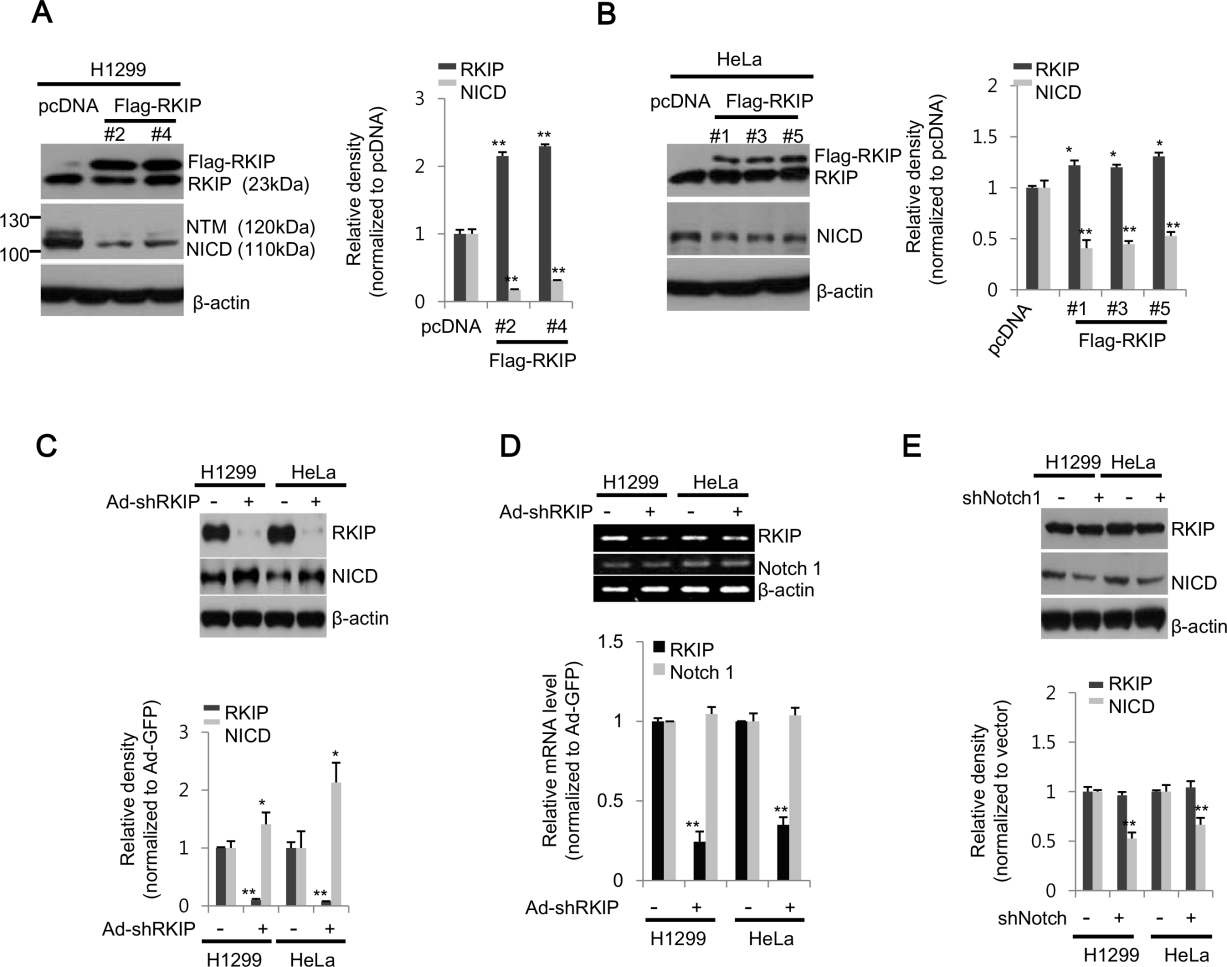
RKIP negatively regulates Notch1 activation in lung and cervical cancer cell lines (**A**, **B**) Stable cell lines expressing FLAG-tagged RKIP (FLAG-RKIP) or control vector (pcDNA) were established in H1299 (clones #2 and 4) and HeLa (clones #1, 3, and 5) cells. (*Left*) Total cell extracts (30 μg) were separated on 10% SDS gels and analyzed by western blot analysis using anti-RKIP or -Notch1 antibodies. β-actin was used as a loading control. (*Right*) The expression levels of RKIP or NICD in H1299 (A) and HeLa (B) cells were quantified and represented graphically. (**C**) In H1299 and HeLa cells, RKIP expression was either knocked down with adenoviral shRNA-RKIP (Ad-shRKIP) or unaffected using a control vector (Ad-GFP). The expression levels of RKIP and Notch1 (NICD) in total cell extracts were analyzed by western blot analysis using specific antibodies to each protein (*top*) and represented graphically (*bottom*). (**D**) Semi-quantitative RT-PCR analysis of Notch1 in RKIP- knocked down H1299 or HeLa cells. PCR products (295 bp for β-actin, 564 bp for RKIP, and 365 bp for Notch1) were separated on a 1.5% agarose gel (*top*) and quantified (*bottom*). (**E**) Expression of Notch1 in H1299 or HeLa cells was either abolished by transient transfection with Notch1 shRNA or unaffected in control vector transfections for 24 h. Each protein was analyzed by western blot analysis (*top*) and quantified (*bottom*). All data indicate the mean values ± standard deviation (S.D.) of at least three independent experiments. *p < 0.05 or **p < 0.01 compared to normalized controls.

Low expression levels of RKIP in tumor tissues are suggestive of poor prognoses in cancer patients, but the functional role of RKIP in cancer metastasis is still poorly defined. To investigate the functional relationship between RKIP and Notch signaling during the migration and invasion of cancer cells, we produced lung (H1299) or cervical (HeLa) cancer cell lines stably overexpressing FLAG-tagged RKIP proteins. Compared to endogenous levels of RKIP, both stable cell lines expressed higher levels of RKIP, but the levels of RKIP in H1299 lung cancer cells were higher than those observed in HeLa cervical cancer cells (Figure 1A, 1B). These RKIP-overexpressing cancer cells showed a similar pattern not only in cell proliferation and cell cycle regulation, but also in cell morphology compared to control cells (Supplementary Figure S1), suggesting that overexpression of FLAG-tagged RKIP does not influence cell growth and proliferation in these cancer cell lines. Interestingly, the levels of NICD, the intracellular activated fragment of Notch1 (110kDa), were significantly decreased in RKIP-overexpressing H1299 cells compared to vector-only (pcDNA3.1) control cells, and similar results were observed in two FLAG-tagged RKIP clones (#2 and 4) (Figure 1A). Also, the NICD levels were similarly decreased when FLAG-tagged RKIP proteins were overexpressed in HeLa cells (Figure 1B). The NTM, a transmembrane fragment of Notch1 (120 kDa), was barely detected in both H1299 and HeLa cells overexpressing RKIP. Conversely, knockdown of RKIP in both H1299 and HeLa cells using a recombinant adenoviral RKIP shRNA-expressing vector (Ad-shRKIP) showed a significant increase of NICD production compared to control cells infected with a GFP-targeted recombinant adenoviral vector (Ad-shGFP) (Figure 1C). Nevertheless, knockdown of RKIP did not affect the mRNA levels of Notch1 in either H1299 or HeLa cells (Figure 1D), suggesting that RKIP-dependent NICD production is likely regulated post-translationally. Lastly, we also knocked down Notch expression using a Notch1 shRNA-expressing vector (pLK 0.1-shNotch1) in H1299 or HeLa cancer cells. Compared to control cells transfected with pLK 0.1-GFP shRNA, expression levels of RKIP were not affected in Notch1-knocking down cells (Figure 1E), suggesting that RKIP is an upstream component of Notch1 signaling in these cancer cells.

### RKIP and NICD exhibit inverse expression patterns in cancerous human cervical and gastric tissues

**Figure 2:**
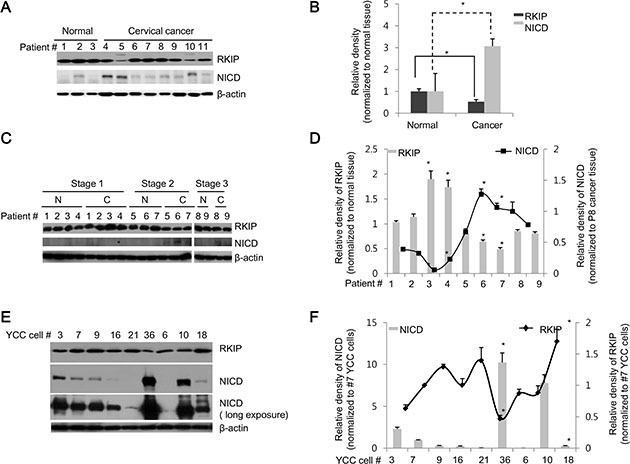
RKIP and NICD are inversely expressed in human cervical and stomach cancer tissues (**A**, **B**) RKIP and NICD expression in human cervical cancer. (A) Proteins were extracted from normal (patients #1–3) and cancerous (patients #4–11) human cervical tissues. Total proteins (30 μg) were subjected to immunoblot analysis. (B) Expression of each protein (RKIP or NICD) was quantified by densitometric scans and represented graphically. *p < 0.05 compared to normalized controls. (C, D) RKIP and NICD expression in human stomach cancer. (**C**) Proteins were extracted from pairs of tumorous and adjacent non-tumorous tissues of nine human stomach cancer patients (patients #1–9) with three different TNM stages (stage 1: patients #1–4, stage 2: patients #5–7, and stage 3: patients #8–9). (**D**) RKIP and NICD expression in stomach cancer specimens (indicated by “*C*”) were quantified followed by normalization to the expression of each protein, respectively, in matched adjacent non-tumorous tissues (indicated by “*N*”) and represented graphically. *p < 0.05 compared to normalized controls. (E, F) Expression of RKIP and NICD in YCC gastric cancer clones (human advanced gastric cancer cell lines). (**E**) Nine YCC clones (#3, 6, 7, 9, 10, 16, 18, 21, and 36) were subjected to immunoblot analyses. (**F**) The expression levels of RKIP and NICD in YCC clones were quantified and represented graphically. β-actin was used as loading control. All data indicate the mean values ± S.D. of at least three independent experiments. *p < 0.05.

As demonstrated above, overexpression of RKIP inhibited the production of NICD in cancer cells. To further assess how the expression of these two proteins is regulated in human cancers, we examined the relative expression of RKIP and NICD in cancer tissues derived from human patients. First, we compared expression levels in normal (three participants) and cancerous (eight patients) cervical tissues and found that RKIP levels were decreased in many cancerous cervical tissues compared to normal cervical tissues. In particular, RKIP levels were significantly lower in some patients (#4, 5, and 10), whereas the expression levels of NICD were highly elevated in these cancerous cervical tissues (Figure 2A). Thus, an inverse relationship exists between the expression levels of RKIP and NICD in human cancerous cervical tissue (Figure 2B).

Second, we compared the expression of RKIP and NICD in paired stomach tissues (tumorous tissue and adjacent non-tumorous tissue) derived from nine patients with three different tumor-node-metastasis (TNM) stages (1–3). Compared to adjacent paired normal tissues, RKIP expression was highly elevated in the tumorous tissues of patients (except patient #1) with TNM stage 1, and NICD was barely detectable in both normal and tumorous tissues within the TNM stage 1 category (Figure 2C, 2D). However, RKIP was somewhat decreased in the tumorous tissues of patients with TNM stage 2 and 3 compared to matched non-tumorous tissues. Conversely, NICD was significantly increased in the tumor tissues found at these stages (Figure 2C, 2D). Lastly, we also examined the expression patterns of RKIP and NICD in highly metastatic Yonsei Cancer Center (YCC) cancer cells, which have been isolated and established from the ascite fluid or peripheral blood of Korean gastric cancer patients (obtained from the Yonsei Cancer Center in Korea). Some YCC clones (#3, 36, and 10) exhibited high expression of NICD but relatively low expression of RKIP compared to other YCC clones, which showed opposite expression patterns (Figure 2E, 2F). Overall, these data suggest that RKIP negatively regulates Notch signaling in human cervical and gastric cancers.

### RKIP inhibits Notch1-mediated invasion and migration of cancer cells

**Figure 3:**
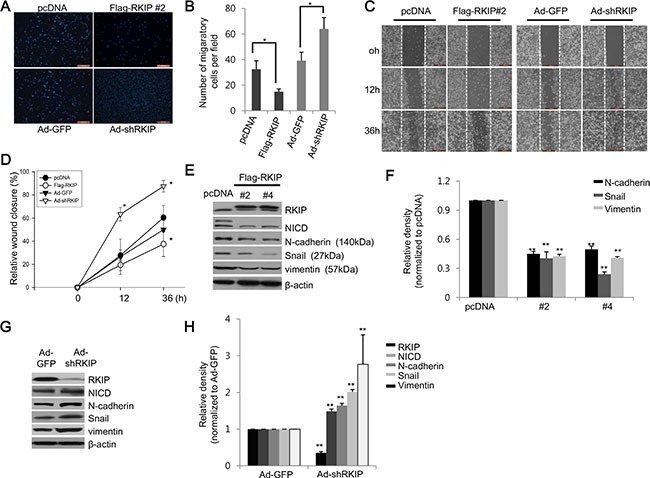
RKIP controls migration and invasion of cancer cells via downregulation of EMT proteins (**A**) The Matrigel invasion chamber assay using RKIP-overexpressing or RKIP-knocking down H1299 cells. Cells that invaded the Transwell insert were stained with propidium iodide and photographed. (**B**) Cells were quantified by counting the cells in ten randomly selected fields under the microscope (10 × objective magnification). *p < 0.05. (**C**) The wound-healing assay using RKIP-overexpressing or RKIP-depleted H1299 cells. Additional assay details described in “Materials and Methods.” Representative images (50 × magnification) indicate cell migration at given times. The wound edge at 0 h is indicated by white (dotted) lines. (**D**) Cell migration represented as relative wound closure (percentage of the average migratory distance of cells relative to the original wound edge). Data indicate the mean values ± S.D. of at least three independent experiments. *p < 0.05 compared to controls. Scale bar, 500 μm. (**E**–**H**) Regulation of EMT-related proteins by RKIP. (E, G) Expression of EMT proteins (N-cadherin, Snail, and vimentin) in RKIP-overexpressing or RKIP-knocking down H1299 cells examined via western blotting. β-actin was used as loading control. (F, H) The relative expression levels of the noted proteins, as determined by densitometric scans, represented graphically. Bars are the mean values ± S.D of three identical experiments. **p < 0.001.

It has been proposed that the loss of RKIP in cancer cells leads to increased EMT. To investigate the possible role of RKIP in cancer, we investigated the invasive and migratory activities of H1299 lung cancer cells when RKIP was either overexpressed or knocked down *in vitro*. RKIP-overexpressing H1299 cells (FLAG-RKIP #2) significantly inhibited cell invasion in Transwell Matrigel assays compared to control H1299 cells (pcDNA) (Figure 3A, 3B). In contrast, ablation of RKIP in H1299 cells highly stimulated *in vitro* cell invasion (Figure 3A, 3B). In addition to cell invasion, we observed similar results in wound-healing assays; overexpression of RKIP inhibited cell migration in a time-dependent manner, whereas RKIP knockdown greatly promoted migration of H1299 lung cancer cells (Figure 3C, 3D).

EMT is a prerequisite for cancer cells to become highly metastatic, and EMT occurs as the result of increased expression of EMT-related proteins, such as N-cadherin, Snail, and vimentin. As such, we tested whether overexpression or knockdown of RKIP could modulate the expression of these EMT-related proteins. As predicted, overexpression of FLAG-tagged RKIP in H1299 cells (clones #2 and 4) significantly reduced expression of Snail, N-cadherin, and vimentin (Figure 3E, 3F), which likely coordinates with a decrease of NICD. In contrast, knockdown of RKIP (Ad-shRKIP) remarkably induced expression of Snail, N-cadherin, and vimentin compared to control H1299 cells (Ad-shGFP) (Figure 3G, 3H).

**Figure 4:**
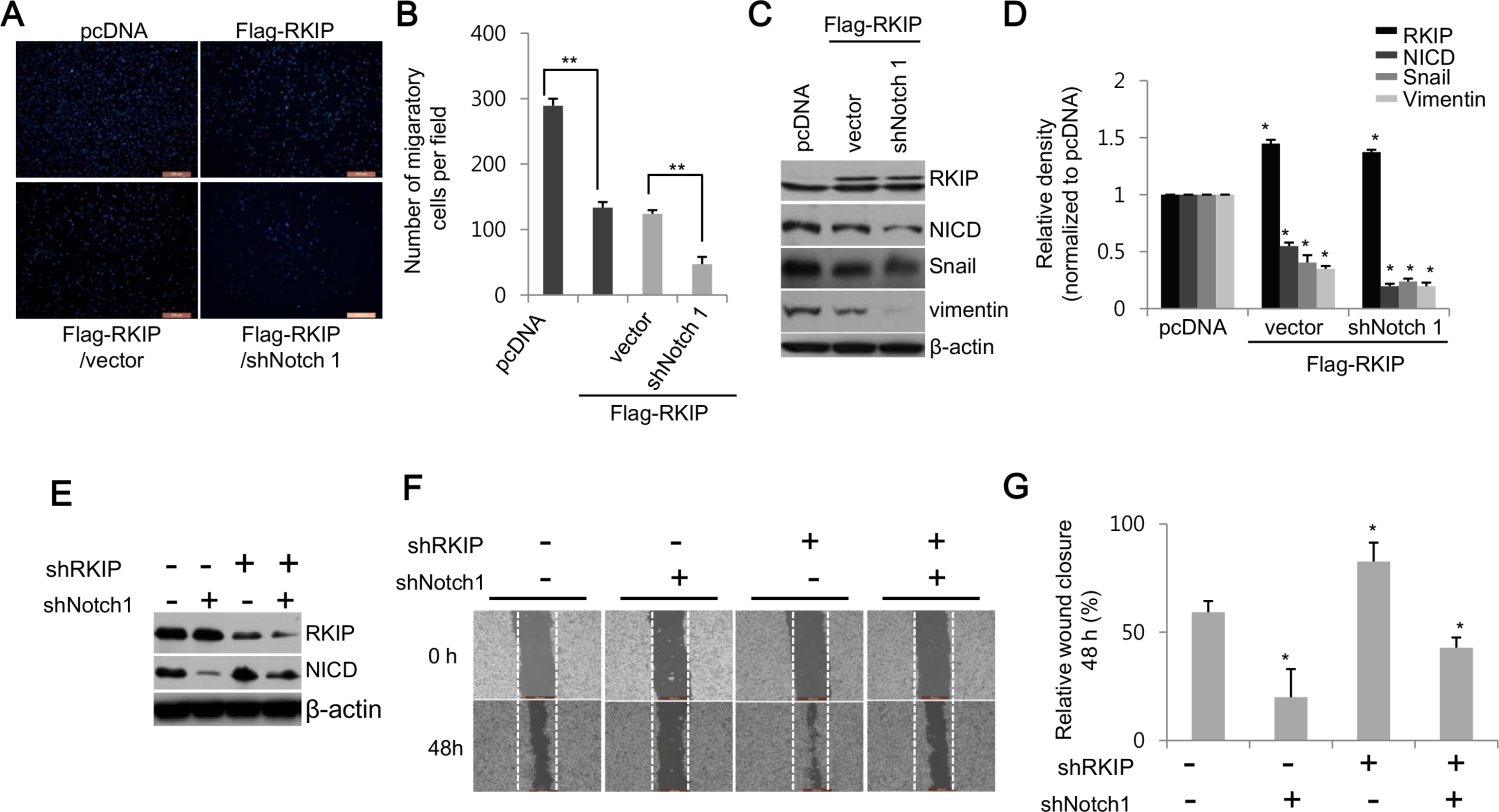
RKIP inhibits cell migration and invasion by directly downregulating Notch1 activity (**A**, **B**) Cell invasion of NICD-depleted, RKIP-overexpressing HeLa cells (FLAG-RKIP #5) was assessed via the Matrigel invasion chamber assay. (A) Photographs of propidium iodide-stained cells that invaded the chamber. (B) Quantification of the number of migratory cells in ten randomly selected fields. **p < 0.001. (**C, D**) Effect of Notch1 ablation on expression of EMT proteins in HeLa cells overexpressing RKIP. (C) Notch1 expression was knocked down by expressing the shNotch1 vector in RKIP-overexpressing HeLa cells (FLAG-RKIP #5), and total protein extracts (30 μg) were subjected to western blot analysis to examine the expression of the EMT markers, Snail and vimentin, in these cells. β-actin was used as loading control. (D) Relative expression levels of these proteins represented graphically. *p < 0.05. (**E–G**) Effect of Notch1 ablation on cell invasion in RKIP-overexpressing HeLa cells. Cell migration analysis of RKIP- and Notch1-knocked down H1299 cells. The expression of both RKIP and Notch1 was knocked down by co-transfecting the shRKIP- and shNotch1-expressing vectors in H1299 cells. (E) Expression of RKIP and NICD in these cells was determined by western blot analysis. (F) Migratory behaviors of these cells were evaluated using the wound-healing assay. (G) Cell migration quantified as described in Figure 3. Data indicate the mean ± S.D of the wound edge closure (%) of monolayer cells compared to controls (0 h). *p < 0.05. Scale bar, 500 μm.

To further confirm whether RKIP acts as an inhibitor of Notch1 activation, we knocked down Notch1 in RKIP-overexpressing HeLa cells (clone #5) in which the NICD expression levels were inhibited up to 50% compared to control HeLa cells (0.55 ± 0.03, *p < 0.005, Figure 1B). Knockdown of NICD via Notch1 shRNA in HeLa cells overexpressing FLAG-tagged RKIP remarkably abolished RKIP-dependent suppression of cell invasion compared to RKIP-overexpressing cells treated with control shRNA vector (Figure 4A, 4B). Furthermore, the decreased production of NICD mediated by Notch1 shRNA in RKIP-overexpressing HeLa cells caused a significant decrease of EMT-related protein expression (Figure 4C, 4D). We also examined the migratory ability of HeLa cells in which both RKIP and Notch1 were simultaneously knocked down. Individually, ablation of RKIP stimulated cell migration in wound-healing assays, whereas knockdown of NICD inhibited cell migration (Figure 3C). However, cell migration in NICD- and RKIP-depleted cells was very similar to that observed during wound closure in non-treated cells; the previously observed increase in cell migration mediated by RKIP knockdown was abolished by the inhibition of Notch1 activation (Figure 4E–4G). These data indicate that RKIP negatively regulates NICD-induced cell invasion and migration in tumorigenesis.

### Loss of RKIP promotes malignant progression of lung tumors *in vivo*

**Figure 5:**
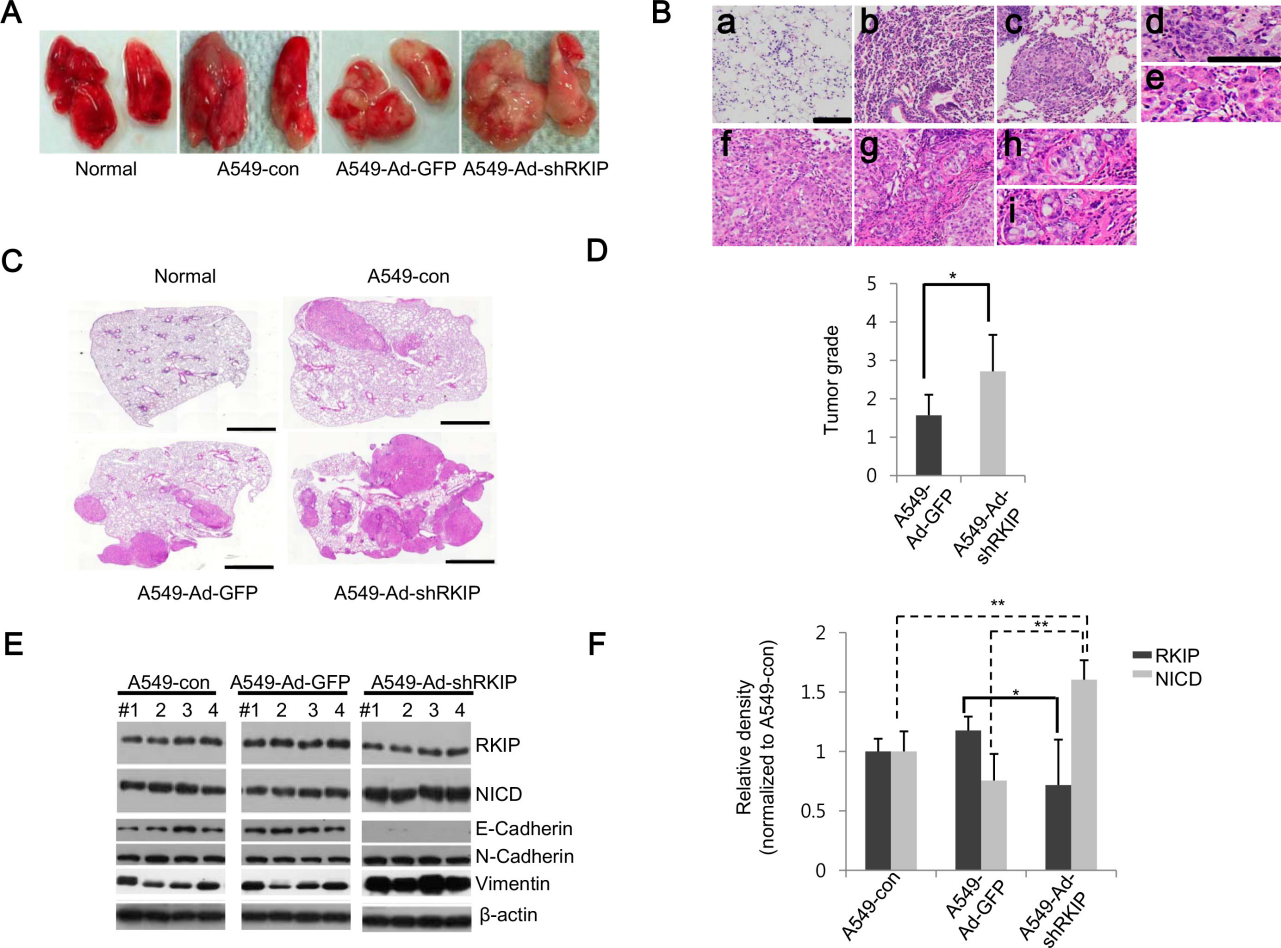
Knockdown of RKIP promotes tumor metastasis *in vivo* in orthotopic lung cancer mouse models (**A**) Images of metastatic lungs. Lungs were extracted from BALB/c nude mice injected with 1 × 10^6^ cells (per mouse) of A549 only (A549-con), A549 cells infected with Ad-GFP (A549-Ad-GFP), or A549 cells infected with Ad-shRKIP (A549-Ad-shRKIP) (*n* = 7 per group) and photographed to visually examine tumor nodules. Normal indicates non-treated mice. (**B**) Four-stage grading systems of lung adenocarcinoma. Based on tumor progression and histopathological phenotypes of H & E-stained tumor tissues, the lung cancer tissues were classified at four stage grades as follows: (a) normal lung tissue, (b) Grade 1 lesions of an atypical adenomatous hyperplasia (AAH) progressing to a small adenoma, (c) Grade 2 adenoma, (d) Grade 2 lesions form a solid tumor but have regular nuclei, (e) Grade 3 adenocarcinoma exhibiting enlarged pleomorphic nuclei and prominent nucleoli, (f) Grade 3 adenocarcinoma displaying mixed cellular phenotypes, (g) Grade 4 invasive adenocarcinoma, (h) Grade 4 adenocarcinoma with glandular/acinar architecture, and (i) Grade 4 adenocarcinoma with desmoplasia. Scale bar, 100 μm. (**C**) Representative H & E-stained lung tissues. Scale bar, 100 μm. (**D**) Distribution of tumor grades. Bars indicate the average values of four grades determined from tumor tissues for each mouse group. Data show mean ± S.D. *p < 0.05 (*n* = 7). (E, F) RKIP and NICD expression in lung adenocarcinoma tissues. (**E**) Total lysates of tumor tissues (four per group) were subjected to immunoblot analysis using the indicated antibodies. (**F**) Quantifications of RKIP and NICD expression levels represented graphically. Data indicate the mean values ± S.D. of three independent experiments. *p < 0.05; **p < 0.01.

To further elucidate the effects of RKIP knockdown on tumor metastasis *in vivo*, we used an orthotopic lung cancer model. We intravenously injected A549 lung cancer cells treated with Ad-shRKIP or Ad-shGFP into the tail vein of BALB/c nude mice and examined the metastatic lesions of the removed lungs after eight weeks. The nude mice transplanted with A549-Ad-shRKIP cells showed more severe tumor nodule formation in the lungs than mice treated with A549-Ad-shGFP cells (Figure 5A, 5C). Furthermore, we employed the four-stage grading system of H & E-stained lung tumor tissues to histologically monitor and quantify tumor development and progression (adopted from ref. 36). The criteria for each grade are as follows: Grade 1 tumors are atypical adenomatous hyperplasia (AAH) or small adenomas that have uniform nuclei (Figure 5B*b*). Grade 2 tumors are larger adenomas that have solid tumor morphologies but slightly enlarged uniform nuclei with prominent nucleoli (Figure 5B*c*, 5B*d*). Grade 3 tumors have cells with enlarged pleomorphic nuclei and nuclear atypia (Figure 5B*e*, 5B*f*). Grade 4 tumors are invasive adenocarcinomas that have glandular/acinar architectures (Figure 5B*g*, 5B*h*) and highly invasive stromal reactions (desmoplasia) to surrounding nests of tumor cells (Figure 5B*g*, 5B*i*). Based on this grading scheme, we confirmed that loss of RKIP (mediated by A549-Ad-shRKIP injections) caused the appearance of severe malignant tumors (average tumor grade: ∼3) in the lung compared to control A549-Ad-shGFP-injected mice (average tumor grade: ∼1.5) (Figure 5C, 5D).

Next, we examined the expression profiles of RKIP and NICD in the lung tumors of these mice. Similar to our *in vitro* results, expression of NICD was significantly elevated in the mice injected with Ad-shRKIP-expressing A549 lung cancer cells compared to control mice, and RKIP expression was relatively less significant because we analyzed these proteins on the total lung tissues that might have contained both normal and cancerous cells (Figure 5E, 5F). Furthermore, N-cadherin and vimentin, markers for EMT, were highly increased in the lung tissues of mice injected with Ad-shRKIP-expressing A549 cells, whereas the expression of E-cadherin was remarkably decreased in these mice (Figure 5E).

### RKIP directly interacts with full-length Notch1 but not NICD

**Figure 6:**
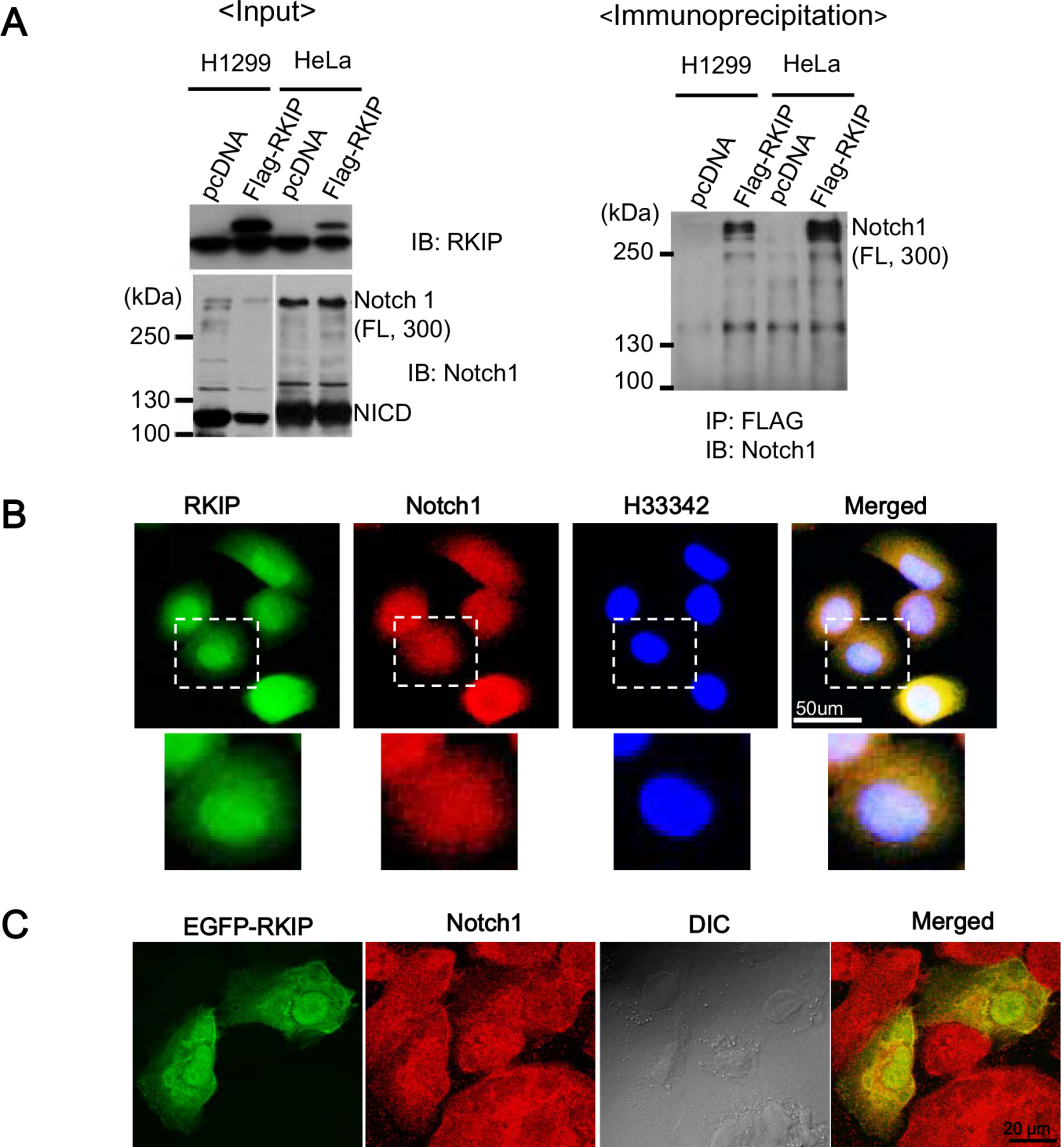
RKIP directly interacts with full-length Notch1 (**A**) The left panel indicates the input of RKIP and Notch1 in FLAG-tagged, RKIP-overexpressing cells. Total cell lysates (1 mg) of FLAG-tagged, RKIP-overexpressing H1299 or HeLa cells and control cells expressing pcDNA were immunoprecipitated (IP) using anti-FLAG antibodies (M2 beads), and the bound Notch1 proteins were detected by immunoblotting (IB) with anti-Notch1 antibodies. (**B**) Co-localization of RKIP and Notch1 using immunohistochemistry. FLAG-tagged, RKIP-overexpressing H1299 cells were fixed, stained with both FITC-conjugated anti-RKIP antibodies and Cy5-conjugated anti-Notch1 antibodies, and visualized under the fluorescent microscope. The nuclei were stained with H33342 dye. White dotted boxed regions correspond to the higher magnification images below. (**C**) Co-localization of EGFP-RKIP and Notch1. EGFP-tagged, RKIP-overexpressing H1299 cells were fixed, stained with both GFP and Cy5-conjugated anti-Notch1 antibodies, and visualized under the fluorescent microscope.

Next, we investigated the physical interaction between RKIP and Notch1 in either H1299 or HeLa cells overexpressing FLAG-tagged RKIP using immunoprecipitation assays. In these cells, we readily detected the NICD fragment of Notch1 (110 kDa) but barely observed full-length (FL) Notch1 (300 kDa) with long exposures (Figure 6A), suggesting that very little FL-Notch1 was present. Using total proteins (1 mg) extracted from cell lysates, RKIP was immunoprecipitated with anti-FLAG antibodies (M2 agarose beads). We found that RKIP specifically bound to FL-Notch1 (300 kDa) and did not associate with NICD fragments (Figure 6A, *right panel*).

Additionally, we investigated whether RKIP and Notch1 colocalize in cells using immunohistochemistry and cells expressing EGFP-RKIP proteins. These studies indicate that RKIP is likely colocalized with Notch1 in the cytoplasm (Figure 6B and 6C), suggesting that, by binding to FL-Notch1, RKIP could prevent the proteolytic cleavage of Notch1 mediated by γ-secretase to produce NICD, which would consequentially decrease the activation of Notch1 signaling. To test this possibility, we examined whether RKIP affects the expression of the γ-secretase complex enzymes, Presenilin-1 and -2, PEN2, and Nicastrin. However, we found that overexpression or knockdown of RKIP in H1299 cells had no effect on the expression of γ-secretase complex enzymes (Supplementary Figure S2).

### Notch1 activation in hypoxic environments is RKIP-dependent

**Figure 7:**
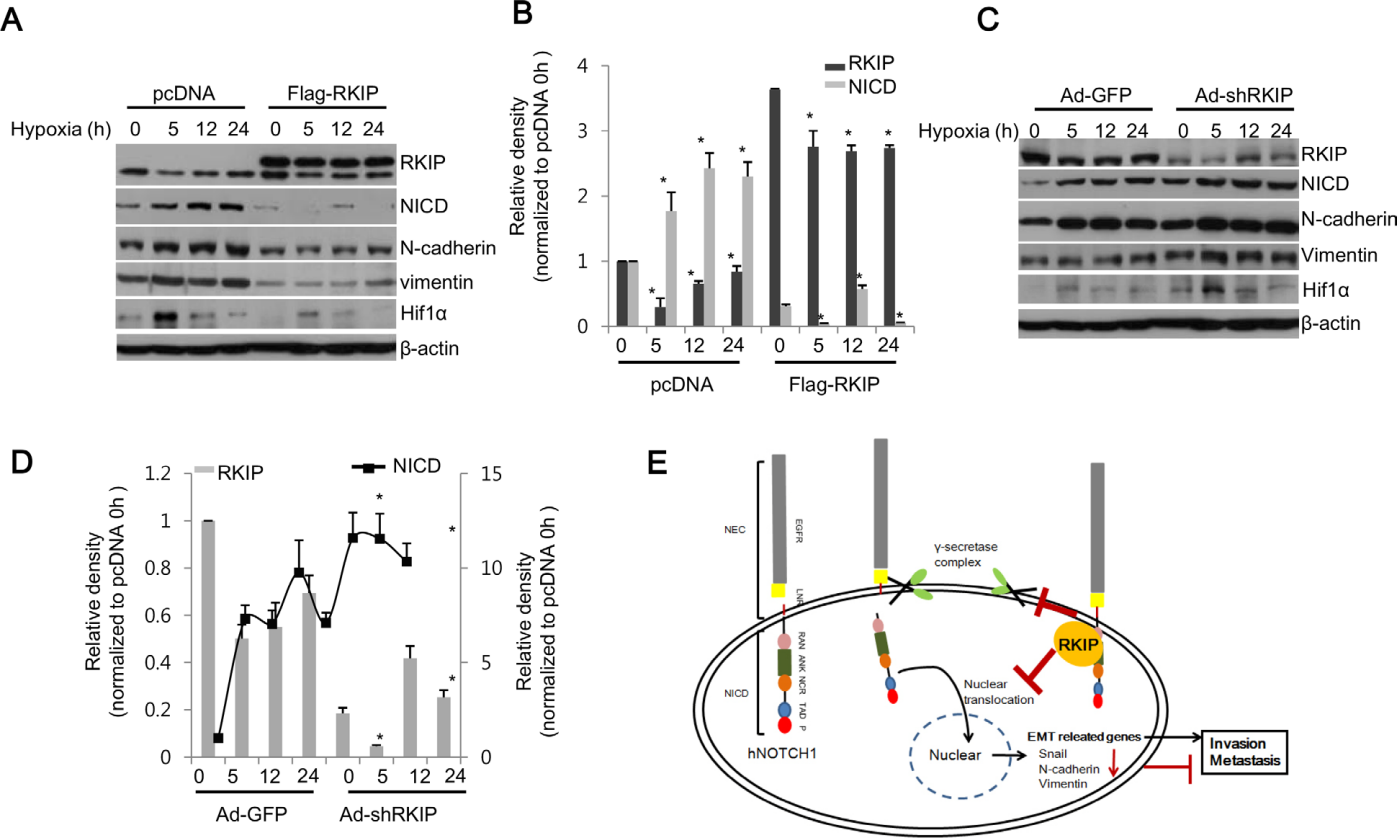
RKIP inhibits NICD-induced EMT in hypoxia (**A**–**D**) RKIP-overexpressing H1299 cells (A, B) or RKIP-depleted H1299 cells (C, D) were incubated under hypoxic conditions (1% O_2_) for the indicated times. (A, C) Total cell extracts (30 μg) were subjected to western blot analysis to examine the expression of each noted protein at the indicated times. (B, D) Expression of each protein was quantified and represented graphically. Data indicates the mean values ± S.D. of three independent experiments. *p < 0.05. (**E**) Schematic illustration of RKIP-mediated regulation of Notch1. *human* Notch receptor 1 (hNotch1) contains two large regions: NEC (Notch extracellular subunits) and NICD (Notch intracellular domain). NICD consists of many motifs, including epidermal growth factor-like repeats (EGFR), Lin12 Notch repeats (LNR), a RAM23 domain (RAM), and Ankyrin repeats (ANK). NICD released by γ-secretase is translocated into the nucleus to activate the translation of EMT-related genes, subsequently promoting cell invasion and metastasis. When RKIP is overexpressed, tumor metastasis is blocked by RKIP-mediated inhibition of Notch1 activation.

According to previous work, Notch1 signaling is highly activated under hypoxia [37, 38]. Here, we tested whether loss of RKIP is necessary for Notch1 activation in hypoxic conditions that assessed by the level of Hif1α. As expected, Notch1 activation (as determined by NICD levels) was gradually increased in a time-dependent manner under hypoxia in normal H1299 cells, but it was significantly inhibited in cells overexpressing FLAG-tagged RKIP (Figure 7A, 7B). This RKIP-dependent Notch1 activation under hypoxia also affected the expression of EMT-related proteins; N-cadherin and vimentin were coordinately elevated with Notch1 activation and conversely inhibited by overexpression of RKIP in hypoxic conditions (Figure 7A, 7B). In contrast, silencing RKIP expression in H1299 cells (Ad-shRKIP) stimulated Notch1 activation under hypoxia, leading to increases of N-cadherin and vimentin compared to control cells (Ad-shGFP) (Figure 7C, 7D). These data demonstrate that hypoxia-induced Notch1 activation is likely due to decreased expression of RKIP.

## DISCUSSION

In the present study we showed that reduction of RKIP expression significantly increased production of an active form of Notch1, NICD, and subsequently promoted the metastatic ability of lung and cervical cancer cells. Conversely, overexpression of RKIP inhibited cell invasion and migration of these cancer cells via downregulation of NICD. These data suggest that RKIP is an important negative regulator of Notch1 activation, which plays a pivotal role in cancer metastasis. Indeed, RKIP has been implicated as a suppressor of metastasis largely because it is significantly downregulated in many types of human metastatic tumors, such as prostate, breast, gastric, colorectal, lung, and cervical cancers. Loss of RKIP in prostate cancer cells shows metastatic phenotypes, and restoration of RKIP expression inhibits prostate cancer metastasis [14–16]. Additionally, the metastatic ability of breast cancer cells positively correlates with reduced levels of RKIP in human cancer cells, closely associating with poor prognosis in breast cancer patients [17, 18]. Survival rates and tumor progression in colorectal, ovarian, and cervical cancer patients are also related to the levels of RKIP in tumor tissues [19–24]. Despite many clinical implications between RKIP expression and cancer metastasis, the molecular mechanism regarding how downregulation of RKIP results in poor outcomes and malignant progression in human cancers is not clear.

RKIP was initially identified as an inhibitor of the Raf/MEK/ERK pathway, which is necessary for growth and proliferation of cancer cells, via its direct interaction with Raf1 [7]. RKIP also stimulates glycogen synthase kinase 3β (GSK3β) signaling by blocking the inhibitory phosphorylation of GSK3β. Subsequently, the activated GSK3β inhibits EMT regulatory proteins, such as Snail or Slug, under a certain conditions [39, 40]. Therefore, RKIP-dependent suppression of the ERK or GSK3β pathways might be associated with the inhibitory role of RKIP in cancer metastasis. Additionally, in this report we suggest that RKIP directly interacts with Notch1 and subsequently prevents the proteolytic cleavage of Notch1 by the γ-secretase complex, diminishing the release of NCID, the activated form of Notch1. Loss of RKIP expression in cancer cells highly stimulated cell invasion and migration and upregulated the expression of EMT-related proteins. Also, some other reports show that diminution of RKIP expression is directly associated with increase of cell growth and motility during cancer metastasis [41, 42]. Together these results suggest that RKIP-dependent activation of the Notch pathway in cancer cells may play a critical role in EMT and metastasis.

According to other work, suppression of NICD proportionally prevents transcriptional activation of EMT-related genes, such as *snail* or *slug*, leading to decreased migration and invasion of tumors [31–33]. Additionally, it has been reported that Notch signaling is frequently upregulated in human malignant tumor tissues, including cervical, lung, colon, gastric, head and neck, prostate, and pancreatic cancers [26–30]. In particular, Notch signaling has been suggested to contribute to the progression of non-small cell lung cancer [34, 35]. In this study, activation of Notch1 (NICD production) in lung and cervical cancer cell lines also promotes tumor invasion/migration and upregulation of EMT-related proteins. Moreover, Notch-dependent metastasis can be stimulated by the reduction of RKIP *in vitro* in cancer cells. Furthermore, the highly increased expression of NICD in human cervical cancer tissues and tumorous tissues of stomach cancer patients with TNM stage 2 is inversely associated with low expression of RKIP in human tumor tissues. However, the role of Notch signaling in cervical cancer is seemingly controversial. Although many cervical tumors are consistently characterized by features associated with misregulation of Jagged1-induced Notch signaling, expression of Notch1 is remarkably reduced or absent in some invasive cervical cancer tissues [43–45]. Overall, Notch signaling plays a critical role in tumor progression and metastasis, and its activation is closely related to RKIP levels in human tumor tissues.

We suggest that RKIP serves as a physiological inhibitor of Notch1 signaling. This raises the question of how RKIP regulates the levels of NICD in tumor cells. In the present study, knockdown of RKIP caused a significant decrease of NICD but no change in the levels of Notch1 mRNA, suggesting that NICD activation is likely regulated post-translationally in an RKIP-dependent manner. Upon activation of Notch signaling, Notch1 is cleaved in a stepwise manner mediated by the proteolytic actions of the γ-secretase complex, leading to the subsequent release of NICD [30]. Therefore, inhibition of γ-secretase would prevent Notch1 activation, resulting in suppression of the Notch signaling pathway. Interestingly, RKIP can directly interact with full-length Notch1 but not NICD. These data suggest that RKIP prevents activation of Notch1 by directly binding to the Notch1 intracellular/transmembrane domain, consequently inhibiting the proteolytic activity of γ-secretase (Figure 7E).

Hypoxia in tumors is closely associated with the potentiation of metastasis via the activation of Notch signaling. According to previous reports, Notch signaling directly modulates the expression of EMT-related proteins by upregulating the expression of Snail and downregulating E-cadherin expression in hypoxic conditions, which stimulates hypoxia-induced tumor cell migration and invasion [37, 38, 46, 47]. Furthermore, Notch signaling stabilizes Snail by stimulating the expression of lysine oxidase (LOX) via recruitment of hypoxia-inducible factor 1α (HIF1α) to the LOX promoter [37]. Therefore, tumor hypoxia appears to be strongly associated with tumor propagation, malignant progression, and resistance to therapy. Consistent with these reports, we found that hypoxia potentiated Notch signaling in H1299 lung cancer cells. However, this hypoxia-induced Notch1 activation was significantly inhibited by RKIP overexpression and stimulated by knockdown of RKIP (Figure 7). Recently, Morecroft et al. (2011) suggested that the lack of RKIP results in exaggerated pulmonary arterial hypertension (PAH) in hypoxia [48]. They also indicated that hypoxia increases activation of the Raf1/ERK pathway by promoting dissociation of RKIP from Raf1. These data support the notion that hypoxia-induced EMT is functionally linked to RKIP expression, which subsequently modulates Notch signaling during metastasis.

In addition to its role as an inhibitor of Notch signaling, demonstrated by the present study, RKIP has been implicated as a suppressor of metastasis via control of the NF-κB/Snail/YY1/RKIP circuitry [49–51]. Hyperactivation of NF-κB in cancer cells transcriptionally activates the expression of Snail and Yin Yang 1 (YY1), and YY1 can also upregulate Snail. The high level of Snail in tumors consequently suppresses the expression of anti-metastatic RKIP. Additionally, NF-kB inhibits RKIP and vice versa. As a result, cancer cells become resistant to chemotherapeutic drugs, leading to high incidences of metastasis and poor outcomes. Furthermore, although molecular links between Notch1 and NF-κB during tumor progression and metastasis have been suggested by the previous work [46, 52], a mechanism of disrupting or directly regulating Notch1 signaling from within the NF-kB/Snail/YY1/RKIP loop was unclear. Our findings indicate that RKIP suppresses activation of Notch1 to inhibit EMT and metastasis, thereby identifying a mechanistic regulatory link between RKIP and Notch signaling.

In conclusion, RKIP, a good marker for cancer prognoses, directly controls the activation of Notch signaling at the post-translational level. Presumably, it blocks the proteolytic activation of Notch1 by γ-secretase, thereby suppressing the production of active NICD. Cancer cells lacking of RKIP expression exhibit more aggressive phenotypes with activation of NICD-induced EMT protein expression. Our findings additionally suggest that the RKIP–NICD signaling axis is not only critical for controlling cancer progression and metastasis, at least in lung, cervical, and gastric cancers, but also for regulating the NF-kB/Snail/YY1/RKIP loop. Therefore, small molecules that mimic the RKIP-dependent inhibition of γ-secretase would be good candidates for the treatment of Notch-induced tumor progression. Furthermore, the identification of such small molecules would provide valuable alternatives to the ineffective γ-secretase inhibitors that fail to pass clinical trials due to dose-limiting toxicities or developments of resistance.

## MATERIALS AND METHODS

### Human tissue samples

Tumorous and adjacent non-tumorous tissues were collected immediately after surgical resection from cervical or gastric cancer patients who were diagnosed with pathologic tumor-node-metastasis (TNM) disease (stage 1–3) at the Hospital of Gyeongsang National University. Tissue specimens were snap-frozen and stored at −80°C. Serial tissue samples were used for protein extraction, stained with hematoxylin and eosin (H & E), and reviewed by pathologists to confirm the diagnoses. This study has been approved by the Institutional Review Board of the Hospital of Gyeongsang National University (IRB #2014–10–024–001, #2012–09–014).

### Cell lines and cell culture

H1299, HeLa, and A549 cell lines were obtained from the American Type Culture Collection. Yonsei Cancer Center (YCC) cell lines were established from the ascites or peripheral blood of patients with advanced gastric cancer by the Cancer Metastasis Research Center at Younsei University College of Medicine (Seoul, Korea). H1299 and A549 cells were maintained in Roswell Park Memorial Institute (RPMI)-1640 medium (Gibco) containing 10% fetal bovine serum (FBS; Gibco). HeLa cells were grown in Dulbecco's Modified Eagle's Medium (DMEM; Gibco) containing 10% FBS. YCC cell lines were maintained in Minimum Essential Medium (MEM; Gibco) supplemented with 1 × non-essential amino acids solution (Gibco) and 10% FBS. All cells were grown at 37°C in a humidified atmosphere of 95% air and 5% CO_2_. For hypoxic treatment, cells were placed in a modulator incubator (Thermo Electron Co.) in an atmosphere consisting of 94% N_2_, 5% CO_2_, and 1% O_2_ for a given time.

### Plasmid constructs, transfection, stable cell lines, and adenoviral vectors

The pcDNA3.1-FLAG-hRKIP plasmid was kindly provided by Dr. Walter Kolch (University of Glasgow, Glasgow, United Kingdom). The shRNA plasmid against human Notch1 (NM_017617) was purchased from Sigma-Aldrich. H1299 and HeLa cells were transfected with plasmids using Lipofectamine2000 (Invitrogen) following the manufacturer's instructions and stably selected in culture medium containing 500 μg/ml G418 (Invitrogen) for two weeks. To generate the RKIP shRNA adenoviral expression vector, oligonucleotide sequences (5′-GGATCCCAAATACAGAGAATG-3′) were cloned using the BLOCK-iT^™^ Adenoviral RNAi kit (Invitrogen) following the manufacturer's instructions. GFP target sequences were used as negative controls. pAd/BLOCK-iT^™^–DEST-RKIP (Ad-shRKIP) and pAd/BLOCK-iT^™^–DEST-GFP (Ad-GFP) plasmids were transfected into 293A cells using Lipofectamine 2000 to obtain viral particles. After 10 days, the viral particles were harvested from the cells and media. The concentrated viruses were used to infect cells. For adenoviral infection, cells were plated in 6-well plates at a density of 1 × 10^5^ cells/mL, infected with adenoviruses at a multiplicity of infection (MOI) of 100, and incubated for 48 h at 37°C.

### Cell invasion assay

A Transwell insert (3422; Corning) with an 8-μm pore size was used to carry out the two-chamber migration assay. The upper surface of the Transwell insert was coated with Matrigel (BD Biosciences; 50 mg/filter), and cells were seeded in a serum free medium. Serum-containing medium (10% FBS) was used in the lower chamber as a chemoattractant. After 20 h, cells that had invaded through the bottom surface of the insert were fixed, stained with propidium iodide (Invitrogen), and photographed. These cells were also quantified by counting the number of cells in 10 randomly selected viewing fields under the microscope (10 × magnification).

### Wound-healing (scratch) assay

Cells were grown to confluence in a 60-mm dish, and a “wounding” line was scratched into the cell monolayer with a sterile 1000-μL pipette tip in three separate places. The width of the wound area was photographed and measured under the inverted phase contrast microscope (Nikon, 50 × magnification) to assess cell migration at 0, 12, and 36 h after scratching.

### RT-PCR

Total cellular RNAs were purified with the TRIZOL^R^ reagent (Life Technology) using the manufacturer's instructions. For RT-PCR experiments, the total RNA (1 μg) was subjected to reverse transcription to produce cDNA. A two-step cycling protocol was used to anneal a pair of primers and elongate DNA synthesis at 55°C. The following forward and reverse primers, respectively, were used: β-actin, 5′-TCA CCC ACA CTG TGC CCA TCT ACG A-3′ and 5′-CAG CGG AAC CGC TCA TTG CCA ATG G-3′; RKIP, 5′-ATG CCG GTG GAC CTC AGC AAG T-3′ and 5′-CGA GCA GCT GTC TGG GAA GTA G-3′; Notch1, 5′-GGC CAC CTG GGC CGG AGC TTC-3′ and 5′-GCG ATC TGG GAC TGC ATG CTG-3′. Each sample was amplified in triplicate. Amplification of β-actin cDNA was used as the endogenous normalization standard. The β-actin, RKIP, and Notch1 PCR products were 295, 564, and 365 bp, respectively. PCR products were analyzed on a 1.5% agarose gel and photographed with the Polaroid Type 667 instant film.

### Western blot analysis

Total proteins were extracted from cell lines and tissues with radioimmunoprecipitation assay (RIPA) lysis buffer supplemented with a protease inhibitor cocktail (Calbiochem). Protein concentration was determined with the Pierce protein assay kit (Pierce). Total protein lysates (30 μg) were separated by SDS-PAGE, and the target proteins were specifically detected by western blotting with antibodies against RKIP (Santa Cruz Biotechnology, sc-28837), Notch1 (Cell Signaling Technology, 3608), cleaved Notch1 (NICD; Cell Signaling Technology, 4147), N-cadherin (Cell Signaling Technology, 4061), Snail (Santa Cruz Biotechnology, sc-28199), vimentin (Santa Cruz Biotechnology, sc-6601), Presenilin-1 (Cell Signaling Technology, 5643), Presenilin-2 (Cell Signaling Technology, 9979), PEN2 (Cell Signaling Technology, 8598), Nicastrin (Cell Signaling Technology, 9447), and β-actin (Sigma-Aldrich, A5441). Proteins were visualized with the Enhanced Chemiluminescence Detection Reagent (Pierce) and quantified using the Sigma-Gel software. Each data was corrected by b-actin and normalized to the control value.

### Tumor metastasis assay *in vivo*

All procedures were conducted in accordance with the National Institutes of Health (NIH) guidelines and approved by the Institutional Animal Care and Use Committee (IACUC) of the Gyeongsang National University (GNU-141119-M0057). For *in vivo* tumor metastasis studies using an orthotopic lung cancer model, BALB/c nude mice (males, six weeks old) were randomly divided into three groups (*n* = 7). A549 cells were infected with recombinant adenovirus expressing RKIP shRNA (A549-Ad-shRKIP) or GFP (A549-Ad-GFP) for 48 h. A549 (A549-con), A549-Ad-shRKIP, or A549-Ad-GFP cells (1 × 10^6^ cells per animal) were suspended in 0.1 mL of sterile phosphate-buffered saline (PBS) and injected into the tail vein of BALB/c nude mice. After six weeks, the mice were sacrificed, and the lungs were extracted. The right lung was directly frozen in liquid nitrogen and stored at −80°C for western blotting, and the left lung was fixed in 10% formalin and embedded in paraffin. Five-micrometer sections were cut and stained with H & E.

### MTT assay

Cell viability of RKIP-overexpressing H1299 or HeLa cells was determined by the methylthiazol tetrazolium (MTT) assay. Cells (2,000 cells per well) were seeded in 96-well plates. After 24, 48, 72, and 96 h, 20 μL of MTT (5 mg/ml) was added to each well. Cells were incubated at 37°C for an additional 4 h. The reaction was terminated by lysing cells with 200 μL DMSO for 5 min. Absorbance was measured at 550 nm in a microplate reader (Hidex). Each experiment was performed in triplicate and repeated three times.

### Flow cytometeric cell cycle analysis

Cells were collected, fixed with ice-cold 70% ethanol for 30 min, and stained with propidium iodide (PI) solution (20 μg/mL of PI and 250 μg/mL of RNase in a solution of 0.1% Triton X-100 in PBS) for 30 min. Cell cycle was analyzed using the EPICS-XL flow cytometer (Beckman Coulter).

### Statistical analysis

Each experiment was conducted independently at least three times, and values were expressed as the mean value ± standard deviation (S.D.). The difference between two groups was assessed by a two-tailed student's *t*-test. Analysis of Variance (ANOVA) statistics were used to compare the means of three groups or more, and these values were verified by the Nonparametric Mann-Whitney *U* test. Values of *p* < 0.05 were considered significant.

## SUPPLEMENTARY MATERIAL FIGURES


