# Supplementary material for: Regulation of the epithelial to mesenchymal transition and metastasis by Raf kinase inhibitory protein-dependent Notch1 activity
Source: Oncotarget. 2015 Dec 22;7(4):4632–46. doi: 10.18632/oncotarget.6728 (PMC4826232; doi:10.18632/oncotarget.6728)
Supplement: Supplementary file 1 [file oncotarget-07-4632-s001.pdf]

Regulation of the epithelial to mesenchymal transition and metastasis by Raf kinase inhibitory protein-dependent Notch1 activity

Supplementary Materials

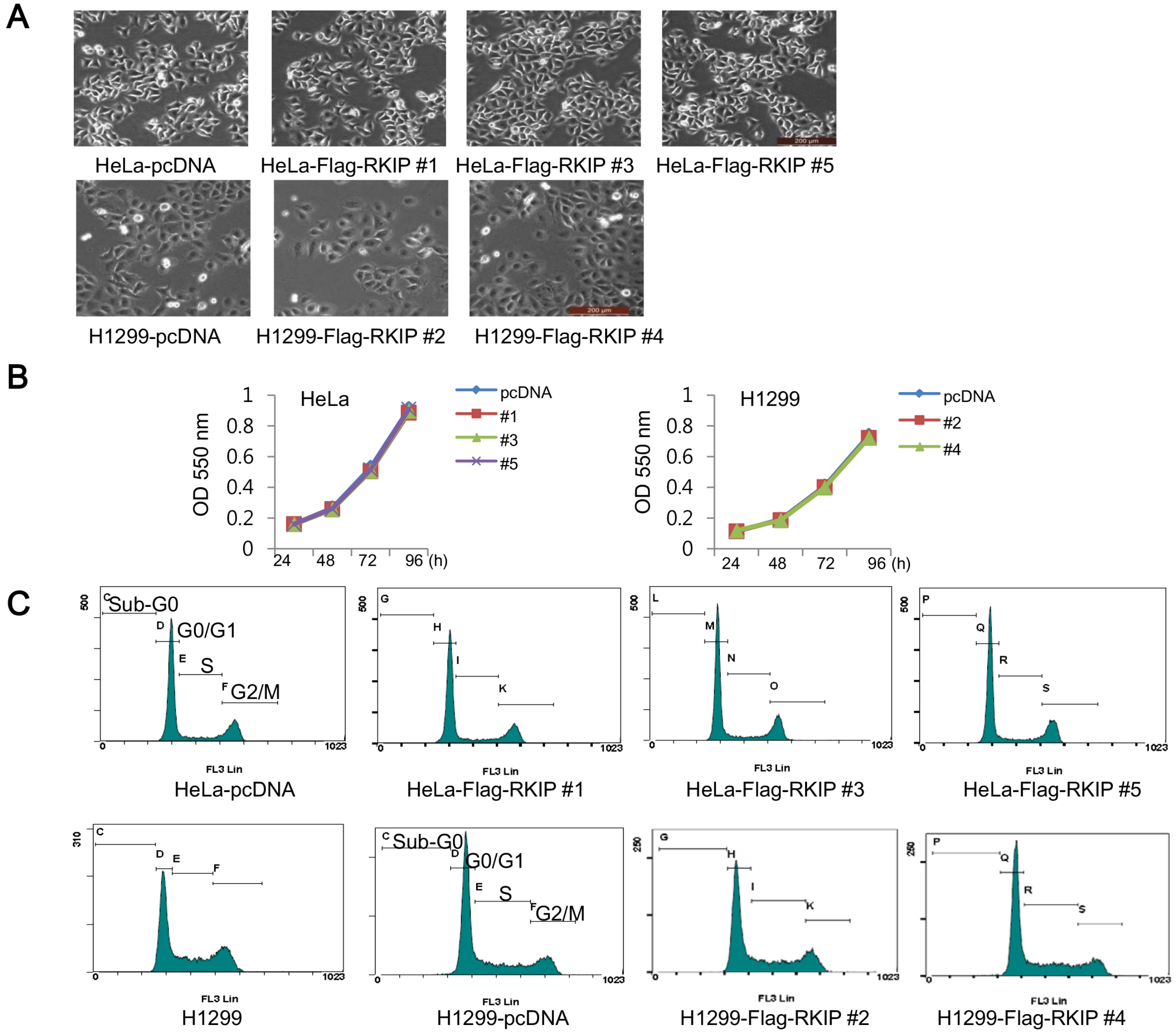

Supplementary Figure S1: Overexpression of RKIP had no effect on cell morphology or cell proliferation. (**A**) FLAG-RKIP-overexpressing HeLa (clones #1, 3, and 5) and H1299 (clones #2 and 4) cells as well as control cells were photographed under the inverted microscope (Leica). (**B**) Cells were cultured in 96-well plates at 2×10^3 cells per well for 24, 48, 72, and 96 h. Cell proliferation of each clone was assessed by the MTT assay. (**C**) The cell cycle of each clone was analyzed by flow cytometery with propidium iodide staining.

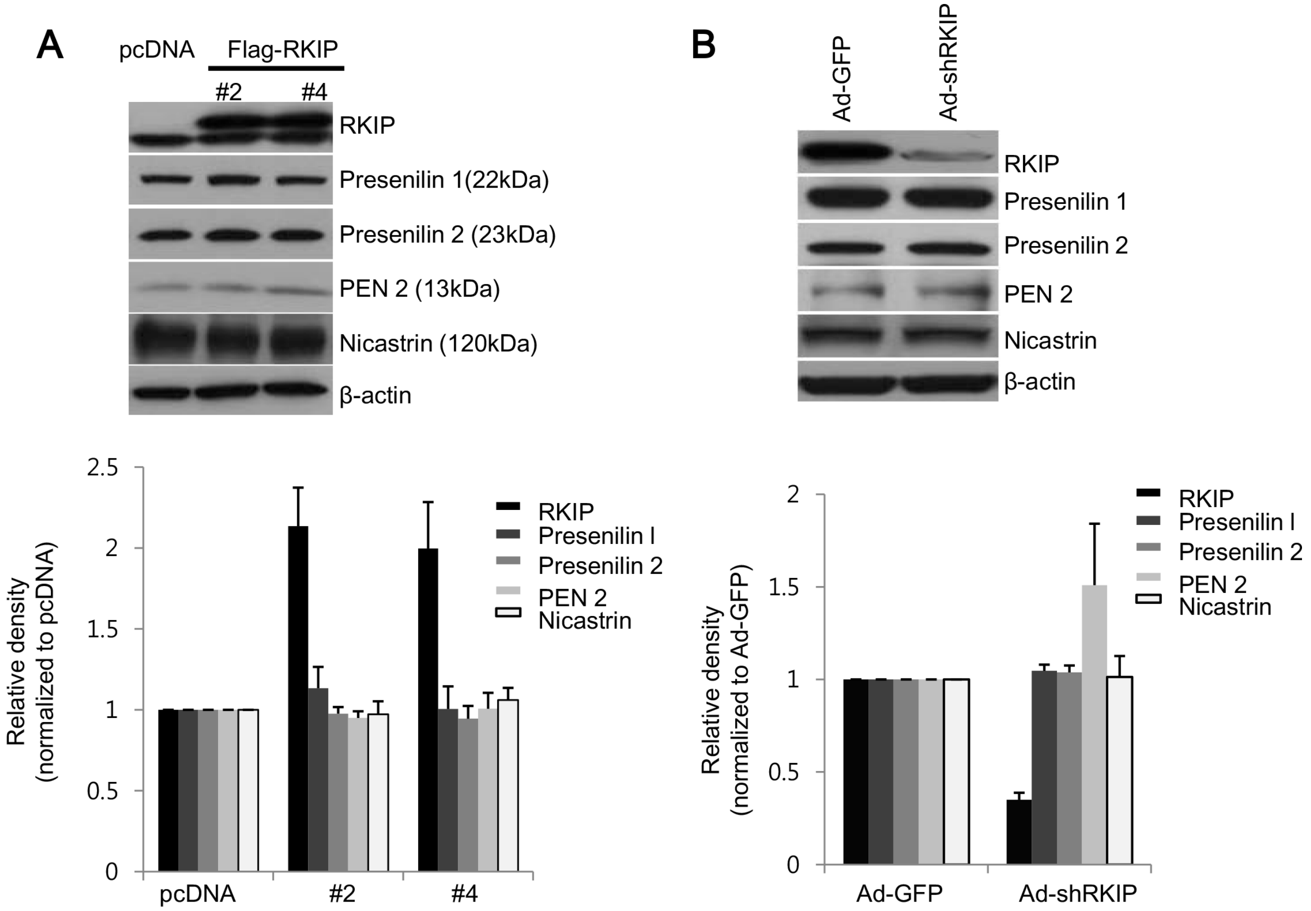

Supplementary Figure S2: RKIP did not influence the expression of γ-secretase complex proteins. (**A**) Expression of γ-secretase complex proteins in RKIP-overexpressing H1299 cells. Total cell extracts of pcDNA-H1299 or FLAG-RKIP-H1299 (clones #2 or 4) were subjected to Western blot analysis using the indicated antibodies (RKIP, Presenilin-1, Presenilin-2, PEN2, or Nicastrin). The expression level of each protein was quantified and represented graphically (*below graph*). (**B**) Expression of γ-secretase complex proteins in RKIP-knocked down H1299 cells. Total cell extracts of H1299 cells infected with Ad-GFP or Ad-shRKIP were subjected to western blot analysis using the indicated antibodies. The expression level of each protein was quantified and represented graphically (*below graph*).
